# Emergent material properties of developing epithelial tissues

**DOI:** 10.1186/s12915-015-0200-y

**Published:** 2015-11-23

**Authors:** Pedro F. Machado, Julia Duque, Jocelyn Étienne, Alfonso Martinez-Arias, Guy B. Blanchard, Nicole Gorfinkiel

**Affiliations:** Department of Genetics, University of Cambridge, Downing Street, Cambridge, CB2 3EH UK; Centro de Biología Molecular Severo Ochoa, CSIC, C/ Nicolás Cabrera 1, Madrid, 28049 Spain; Université Grenoble Alpes, Laboratoire Interdisciplinaire de Physique, BP 53, Cedex 9, Grenoble, 38041 France; CNRS, Laboratoire Interdisciplinaire de Physique, BP 53, Cedex 9, Grenoble, 38041 France; Department of Physiology, Development and Neuroscience, University of Cambridge, Downing Street, Cambridge, CB2 3DY UK

**Keywords:** Viscoelastic fluid, Mechanical properties, Apical contraction, Actomyosin, Oscillations, Hysteresis

## Abstract

**Background:**

Force generation and the material properties of cells and tissues are central to morphogenesis but remain difficult to measure in vivo. Insight is often limited to the ratios of mechanical properties obtained through disruptive manipulation, and the appropriate models relating stress and strain are unknown. The *Drosophila* amnioserosa epithelium progressively contracts over 3 hours of dorsal closure, during which cell apices exhibit area fluctuations driven by medial myosin pulses with periods of 1.5–6 min. Linking these two timescales and understanding how pulsatile contractions drive morphogenetic movements is an urgent challenge.

**Results:**

We present a novel framework to measure in a continuous manner the mechanical properties of epithelial cells in the natural context of a tissue undergoing morphogenesis. We show that the relationship between apicomedial myosin fluorescence intensity and strain during fluctuations is consistent with a linear behaviour, although with a lag. We thus used myosin fluorescence intensity as a proxy for active force generation and treated cells as natural experiments of mechanical response under cyclic loading, revealing unambiguous mechanical properties from the hysteresis loop relating stress to strain. Amnioserosa cells can be described as a contractile viscoelastic fluid. We show that their emergent mechanical behaviour can be described by a linear viscoelastic rheology at timescales relevant for tissue morphogenesis. For the first time, we establish relative changes in separate effective mechanical properties in vivo. Over the course of dorsal closure, the tissue solidifies and effective stiffness doubles as net contraction of the tissue commences. Combining our findings with those from previous laser ablation experiments, we show that both apicomedial and junctional stress also increase over time, with the relative increase in apicomedial stress approximately twice that of other obtained measures.

**Conclusions:**

Our results show that in an epithelial tissue undergoing net contraction, stiffness and stress are coupled. Dorsal closure cell apical contraction is driven by the medial region where the relative increase in stress is greater than that of stiffness. At junctions, by contrast, the relative increase in the mechanical properties is the same, so the junctional contribution to tissue deformation is constant over time. An increase in myosin activity is likely to underlie, at least in part, the change in medioapical properties and we suggest that its greater effect on stress relative to stiffness is fundamental to actomyosin systems and confers on tissues the ability to regulate contraction rates in response to changes in external mechanics.

**Electronic supplementary material:**

The online version of this article (doi:10.1186/s12915-015-0200-y) contains supplementary material, which is available to authorized users.

## Background

The generation of forces and the response to shape deformations are key elements in morphogenesis, which, at the mechanical level, crucially depend upon the material properties of cells and tissues and the underlying cytoskeletal activity. Although much is known about the mechanical properties of isolated cells and monolayers [1, 2], the mechanical properties of developing tissues remain significantly less explored [3]. Experiments with isolated cells have shown that cell mechanical properties greatly depend on the timescale over which these properties are measured. Cell rheology experiments have indicated the presence of distinct regimes of mechanical response to perturbations at very short (10^−3^– 1 s), short (1–10 s) and intermediate (10– 10^3^ s) timescales [4–6], while creep and stress relaxation studies have highlighted the relative contributions of elastic and viscous effective responses of cells to perturbations across time [7, 8]. In addition, cells have been known to modulate their mechanical properties according to their microenvironment [2], and measurements of cultured cell monolayers have shown that the material parameters of monolayers differ significantly from those of isolated cells [9]. These responses are the composite effect of the typical timescales associated with internal and external stresses, the turnover of cytoskeletal proteins and relaxation times of cytoskeletal networks, and the regulation of intercellular adhesion, among other factors. Thus, an important open question in development is: What is the emergent mechanical response of a tissue’s constituent cells at timescales relevant to morphogenesis? This challenge is compounded by the fact that cells are active materials, with the activity of cytoskeletal proteins both driving shape deformation and conferring on cells their emergent material properties [2, 10]. Understanding the interplay between cytoskeletal force generation and associated mechanical response and its integration at the cellular and tissue levels is thus a central issue in cell biomechanics.

The measurement of material properties of cells in living tissues poses a significant technical challenge. Common techniques such as laser ablation [11] and others such as atomic-force microscopy, micro-aspiration and microbead manipulation, laser tweezers [12] and gel-based force sensors [13], can all derive relative or absolute mechanical properties of great interest. However, each technique also comes with its assumptions and limitations, such as the disruption caused to the morphogenetic process, calibration issues, whether perturbations are physiologically relevant [3, 9], and the fact that these are difficult techniques that often produce variable estimates. A complementary set of approaches are non-invasive, using reverse inference methods to extract mechanical properties from cell geometries [14–16]. Though on their own these inference methods cannot extract absolute mechanical properties, their strength lies in the sub-cellular spatial detail and, if performed on time series, in the detailed continuity of mechanical estimates. Combinations of continuous inference methods calibrated by mechanical intervention would be most powerful.

Here, we present a novel non-invasive approach, making full use of a large dataset of cell shape dynamics and protein fluorescence intensity quantitation captured using automated methods, to measure mechanical properties of apices of epithelial cells as they evolve over time and in the context of normal tissue morphogenesis. As a model system, we have used the amnioserosa tissue of the *Drosophila* embryo. The amnioserosa is a squamous epithelium that provides a major driving force to dorsal closure [17], a morphogenetic process during late *Drosophila* embryo development whereby an epidermal gap, bridged by the amnioserosa, is closed to generate epidermal continuity [18]. This closure is effected through the apical contraction of individual amnioserosa cells, which reduce their area in a pulsatile manner via the periodic assembly and disassembly of medial actomyosin foci, with oscillation periods in the range 90–360 s [19–21]. Laser ablation experiments have established ratios of mechanical properties and a transition towards more solid-like behaviour in amnioserosa cells as dorsal closure progresses [22], but how insights from ablation relate to the active contractile forces in the system and how they reflect on the effective material properties of the tissue remain crucial unexplored issues.

Taking myosin fluorescence intensity as a read-out for active cellular force and quantifying cell area deformation in terms of apical strain, we have analysed these data as an experiment of mechanical response under cyclic loading [23] and determined the evolution of the material parameters of the tissue throughout dorsal closure. We show that amnioserosa cells behave as a viscoelastic fluid at timescales relevant for tissue morphogenesis, with cells becoming stiffer and transitioning to a more solid-like behaviour as dorsal closure progresses. Combining our findings with those from previous laser ablation experiments [22], we show that all of medial and junctional stress, and emergent stiffness increase over time, with the most marked increase for apicomedial stress, which quadruples. Finally, we made use of embryos in which myosin phosphorylation is increased and extracted the mechanical properties of the amnioserosa using the same framework. We find that the tissue becomes stiffer and more solid-like compared to wild type, which further validates our framework as a useful method to obtain unambiguous mechanical properties in tissues undergoing oscillatory behaviour.

## Results

**Fig. 1:**
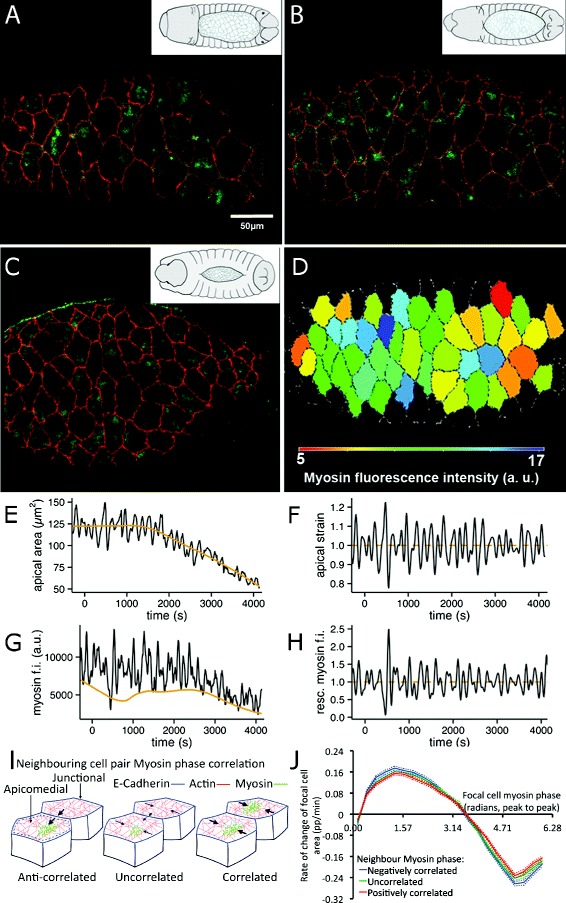
Live imaging and cell area and myosin measurements. **a–c** Confocal dorsal projections of amnioserosa tissues carrying membrane (DECadGFP, *red*) and myosin (zipperYFP, *green*) markers during early (**a**), slow (**b**) and fast (**c**) dorsal closure. *Insets* represent dorsal views of *Drosophila* embryos for the corresponding dorsal closure stages. **d** Segmentation of amnioserosa tissue, with segmented cells coloured according to myosin fluorescence intensity levels. **e** Apical area and **g** myosin fluorescence intensity evolution for a sample cell in the dataset. The area trend and myosin minima trends are shown in *orange*; time *t*=0 corresponds to the onset of slow dorsal closure. Resulting **f** apical strain and **h** rescaled myosin for the raw signals (**e**) and (**g**), respectively. **i** Cartoon showing three classes of relative myosin phase correlation used in the neighbour analysis. *Arrows* show the specific effect of neighbour myosin phase, all other things being equal, on the movement of membranes, which is the outcome of the balance between the relative contractile forces within each cell. The apicomedial region within which myosin fluorescence intensity is measured, excluding junctional myosin, is shown enclosed by the *grey dashed line in cell in left-hand cartoon*. **j** For all fluctuating cells that have a fluctuating neighbour, the dependence of the rate of change in area of the focal cell on its myosin phase is shown, broken down by the relative myosin phase of the focal cell and its neighbour. Relative myosin phase is classified as correlated, uncorrelated or anti-correlated (see also ‘Methods’ and Additional file 4: Figure S4). *Dotted lines* show 95 % confidence intervals

During the approximately 3 hours spanning dorsal closure, the amnioserosa can be characterised at the tissue level by three developmental phases (Fig. 1a–c), early dorsal closure, which lasts approximately 45 min from the end of germ-band retraction; slow dorsal closure, determined by the onset of net tissue area reduction and defined as starting at time *t*=0 min; and fast dorsal closure, starting at ca. *t*= 80 min after the onset of net area reduction [24]. For single cells, dorsal closure evolution is characterised by an attenuation in the amplitude of apical area oscillations and an increase in the average dominant oscillation frequency over time [19, 25]. Genetic perturbation and live imaging experiments have shown that amnioserosa cell area oscillations are driven by transient myosin II motor foci (Fig. 1a–c), which coalesce and disperse over the course of an oscillation period in anti-correlation with apical area [19, 20, 26].

Although relative levels of myosin activity and the duration of myosin foci have been previously measured in dorsal closure [19, 20, 27], a detailed quantitative analysis of myosin fluctuations over the whole process has been lacking. We have thus generated hour-long time-lapses of the amnioserosa using both a membrane marker (DECadGFP) and a myosin reporter (zipperYFP) with a 10-s temporal resolution, allowing us to measure apical cell shape and myosin fluctuations throughout dorsal closure (Fig. 1d–h, Additional file 1: Figure S1, Additional file 2: Figure S2 and see ‘Methods’). To extract these measurements, we defined thin surfaces that cut through the apices of cells and which follow the curvature of the amnioserosa in the embryo, and performed semi-automated tracking of amnioserosa cells on these layers [28]. We measured the apical shape changes of cells from the tracked cell contours. In amnioserosa cells, apical myosin localises both at the apical surface, forming transient accumulations, and at cell–cell junctions. We observed that junctional myosin levels are low compared to apical myosin and do not show consistent fluctuations (data not shown). Thus, we focused our measurements on the medial myosin population. For the same layers in the myosin channel, we calculated medial myosin fluorescence intensity levels from the greyscale value of all pixels in a custom-defined apical surface area in which the membrane region was excluded (see ‘Methods’). Our dataset thus consisted of quantified apical cell shape strain rates and apicomedial myosin density measures for *N*_cells_=675 with a mean track length of 2200 s at a 0.1-Hz sampling rate.

### Rescaled myosin fluorescence intensity and apical area strain

Amnioserosa cell area measurements can be decomposed into two parts, a long-timescale area trend around which oscillations occur and which decreases over time due to net area contraction, and the oscillatory part (Fig. 1e). Since we are interested in area and myosin dynamics at the timescales of the oscillations, we extracted the oscillatory contribution from individual cell areas by computing the area trend via a moving boxcar average and subtracting this from the raw area measurements. Normalising the oscillatory contribution by the area trend and applying a bandpass filter to remove high-frequency noise and low-frequency residuals, we obtained an apical cell area strain, which provides a measure of relative cell area deformation (Fig. 1f).

For the myosin signal, we observed that the minima in the fluorescence intensity measurements are non-zero and decrease over time (Fig. 1g). That myosin fluorescence intensity levels are non-zero can be attributed to both background fluorescence and the possible contribution of a non-oscillating myosin population. The decrease of the myosin signal over time, on the other hand, is a consequence of photobleaching (see ‘Methods’ and Additional file 3: Figure S3). In a similar procedure to the one used to measure cell area oscillations, we computed the trend in myosin fluorescence intensity minima via a moving boxcar average. Because at this stage we are interested in the oscillatory component of the myosin signal, the myosin fluorescence intensity minima trend was subtracted from the total myosin fluorescence intensity measurements. In this way, photobleaching effects are accounted for and the possible contribution of a non-oscillating myosin fluorescence signal is removed. Applying a bandpass filter to the resulting signal to remove high- and low-frequency noise, we obtain the rescaled myosin fluorescence intensity, which provides a measure of the relative myosin activity in individual cells (Fig. 1h). This rescaled myosin fluorescence intensity, together with apical area strain, constitute the fundamental biological measurements going into our analysis (see ‘Methods’ for further details).

To analyse the relationship between cell shape oscillations and myosin dynamics, we asked whether a cell’s area deformation depends exclusively on its own myosin signal or whether the myosin status of neighbouring cells also makes a significant contribution to its deformation. The phase and orientation of cell shape fluctuations are known to be transiently coordinated into characteristic lines or ribbons of cells that are in phase [19], though it was not clear whether or how myosin status was coordinated. We therefore looked closer at the rate of cell shape change in neighbouring pairs of cells of different relative myosin phase, classifying neighbour cell pairs as positively correlated (myosin phase correlation, *r*_m_>0.33), uncorrelated (−0.33≥*r*_m_≥0.33) or anti-correlated (*r*_m_<−0.333) (Fig. 1i). Considering first the cell shape strain rates in the orientation of the vector between neighbouring cell centroids, cell pairs with anti-correlated myosin phases have almost twice the amplitude of strain rate compared to positively correlated pairs (see ‘Methods’ and Additional file 4: Figure S4A). This implies that in-phase cell neighbours compete while anti-phase neighbour facilitate each other’s shape change. However, when considering the effect of neighbours on the rate of whole cell area change, the dependence on the relative myosin phase of neighbours almost completely disappears (Fig. 1j), reducing to an effect largely indistinguishable from randomised data in which neighbouring cells have been replaced by cells selected at random from the rest of the tissue (Additional file 4: Figure S4B). Thus, though cell shape change in the orientation of a neighbour is directly affected by that neighbour’s myosin status, this effect is accommodated in the perpendicular orientation, suggesting that the apical area strain rate provides a good estimate of the intrinsic response of a cell to its myosin phase.

### Time evolution of strain and myosin oscillations

**Fig. 2:**
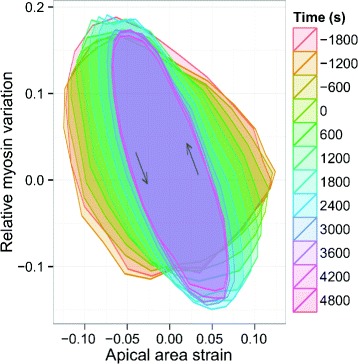
Linear response of apical strain under cyclic myosin loading. Strain–myosin loops across dorsal closure development, obtained by averaging the apical strain and myosin signals of individual cells over the instantaneous oscillation phase defined by the strain signal. *Arrows* indicate the direction of the cycle. We note that the loops are hysteretic and become elliptic, indicating a linear viscoelastic relation between strain and myosin

Having shown that a cell’s apical area deformation is mostly dependent on its own myosin signal, we next investigated the relationship between myosin and area strain. Using strain amplitude as the reference oscillatory signal, we determined the instantaneous phase of individual cell tracks according to their relative position in the oscillation cycle. Binning our data in 10-min intervals and averaging over the instantaneous phase, we then computed the average apical strain and myosin amplitude across cycles over dorsal closure development. Plotting these two averages versus each other, we obtain the strain–myosin loops shown in Fig. 2. We observe that the strain–myosin loops are hysteretic, in accordance with the fact that peaks in myosin fluorescence intensity generally precede troughs in cell area, as reported in [19], and we note that the loops become tighter as dorsal closure evolves, which suggests that the lag between the two signals decreases over time. Furthermore, we note that the loops become more inclined over dorsal closure evolutions, which suggests a higher decrease in the amplitude of strain oscillations across time compared with myosin amplitude. Lastly, we note that the shape of the loops becomes elliptic, which suggests the relation between the two signals can be approximated as linear (see Additional file 5: Figure S5 for examples of non-linear strain–myosin loops).

**Fig. 3:**
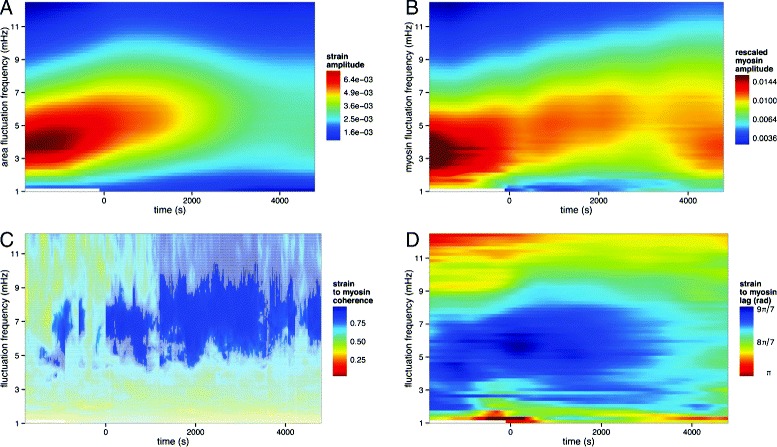
Time evolution of strain and myosin oscillations. Spectrograms of **a** strain amplitude and **b** rescaled myosin amplitude averaged over our dataset. The time evolution of individual frequency contributions were computed via a sliding time window with frequency-dependent length followed by a Fourier transform. The dominant amplitude contributions for strain and myosin correspond to oscillation periods ranging from ca. 3.8 mHz (285 s) in early dorsal closure to 6.5 mHz (153 s) in late dorsal closure. **c** Average cross spectral coherence of area strain and myosin signals. The *whitened region* corresponds to values below the 90 % confidence interval, calculated via a hierarchical bootstrap algorithm. **d** Phase difference between the strain and myosin signals at each frequency component across time, representing the lag of the strain signal with respect to the myosin signal relative to the oscillation period

More quantitatively, the oscillatory behaviour of the strain and myosin signals naturally lends itself to the use of Fourier transform methods for their analysis. Using a sliding time window with frequency-dependent length and applying a Fourier transform, we have investigated the time evolution of individual frequency contributions to the myosin and strain time series of individual amnioserosa cells in the 1–12-mHz range. Averaging over all cells in our dataset, the resulting spectrograms for relative myosin and strain amplitude contributions over time are shown in Fig. 3. We observe a transition towards higher frequency contributions as well as a gradual, general decrease in strain amplitudes over time (Fig. 3a), in agreement with previous reports [19, 25]. In contrast, while the dominant mode contributions to the myosin amplitude also shift towards higher frequencies over time (Fig. 3b), we observe a far less marked attenuation beyond *t*≃0 s, with amplitudes remaining relatively high and constant over the whole course of dorsal closure.

To assess the relation between the two signals across frequencies and time, we have computed their average spectral coherence with a wavelet decomposition (Fig. 3c). Our computations reveal markedly high values for the strain-to-myosin coherence, which further increase as dorsal closure progresses. These results further suggest that the relationship between rescaled myosin and strain is consistent with a linear behaviour.

Previous analysis of average cell area and myosin oscillations has shown that the two quantities are anti-correlated [19]. Computing the phase difference between the individual frequency components of the strain and myosin signals across time and averaging over cells, we obtain the time–frequency phase difference plot shown in Fig. 3d. We observe the expected anti-correlation between the strain and myosin signals at the level of individual frequency components (*π*≤lag≤3*π*/2). Moreover, we observe a reduction in the phase difference over time.

Our results show that the observed attenuation in strain amplitude across time is not matched by a similar attenuation in the myosin amplitude. In addition, there is a reduction in strain-to-myosin lag, together suggesting a change in amnioserosa biomechanics as dorsal closure progresses. In particular, this could be due to an increase in tension in the tissue at the oscillation timescale (so that more of the active myosin force is used to counter resistive forces), a change in the material properties of cells, or both. We will decouple these scenarios in the next sections.

### Linear hysteretic response of apical strain under cyclic loading

Our goal is to determine the emergent material parameters of amnioserosa cells. A common way to probe the mechanical properties of a material in rheology is a cyclic-loading experiment, whereby a sample is subjected to a sinusoidal strain (or stress) input and its stress (strain) response is measured. In analogy with such cyclic-loading experiments and exploiting the oscillatory nature of amnioserosa apical cell strain and myosin signals, we wish to determine the stress–strain response of individual amnioserosa cells across time.

To proceed, we need to determine how cell area strain and cell myosin combine to generate apical stress, by establishing a constitutive equation. To this effect, we make use of three sets of observations. Firstly, our analysis of neighbour effects suggests that the intrinsic response of a cell to its myosin activity is adequately captured by its area deformation. To a first approximation, we can therefore neglect neighbour effects. Secondly, the observed strain-to-myosin coherence (Fig. 3c) suggests that the relationship between these two quantities can be approximated as linear. We also note that amnioserosa cell shape changes occur at low strain rates [19, 25, 29] and that the observed range of area and myosin oscillation frequencies spans only a single order of magnitude. These features allow us to approximate the mechanical behaviour of amnioserosa cells by that of a linear viscoelastic material with a single relaxation timescale. Thirdly, we note that biomechanical models exploring the long-term net contractile behaviour of amnioserosa cells suggest the cells have no memory of an intrinsic equilibrium area [30, 31], while holographic laser ablation used to isolate single amnioserosa cells indicates that amnioserosa cells are under very low elastic strain and that this is incompatible with a high-strain model [29]. Motivated by these findings, we therefore neglect direct strain contributions in our linear viscoelastic approximation. Lastly, we assume that cells are in mechanical equilibrium with their neighbours and that the viscous drag exerted by the yolk and perivitelline fluid is very small compared to morphogenetic forces [32].

Combining all these elements, we arrive at the following constitutive equation for amnioserosa cells relating cell stresses to strain rates and myosin-driven forces,

(1) $$ \sigma + \tau_{\mathrm{R}} \dot\sigma = \bar\tau \kappa c_{\mathrm{m}} + \tau_{\mathrm{R}} \kappa \dot\varepsilon,  $$

where *σ* is the local stress, *c*_m_ is myosin concentration quantified in terms of rescaled myosin fluorescence intensity, and $\dot \varepsilon $ is the apical area strain rate (see ‘Methods’). Our constitutive equation depends on three parameters: *τ*_R_, which denotes the stress relaxation time in the absence of myosin activity, *κ*, which denotes the bulk cell elastic modulus, and $\bar \tau $, which represents the ratio of stress relaxation to active force generation timescales. This equation describes the behaviour of a linear viscoelastic fluid in the presence of a source term, the myosin-driven contractile stress $\bar \tau \kappa c_{\mathrm {m}}$. We note that, even in the absence of an explicit strain term, this myosin term endows the material with an effective equilibrium area, determined by the balance of forces between myosin contractility, stress felt by the cell and cell area strain rate [33]. As a result, this equation describes the behaviour of an effective linear viscoelastic solid. Using this constitutive equation together with strain and myosin measurements, we can determine the associated emergent stress–strain response.

### Emergent material properties of amnioserosa cells

The stress–strain response of a linear material can be parameterised in terms of a complex modulus *E*^∗^(*ω*) relating stress and an oscillatory strain of given frequency *ω*,

(2) $$ E^{*} = E \mathrm{e}^{\mathrm{i}\delta} = \frac{\sigma(t)}{\varepsilon_{0} \mathrm{e}^{\mathrm{i}\omega t}},  $$

where the stiffness *E* quantifies the deformability of the material under applied load and is given by the ratio of stress to strain amplitudes. The phase difference *δ* between stress and strain, commonly expressed in terms of the loss tangent tan*δ*, quantifies the balance between lost and stored energy under an oscillation cycle. We again exploit the oscillatory nature of the strain and myosin signals in amnioserosa by decomposing the cell measurements into the relative amplitudes and strain-to-myosin lags at the individual frequencies set by their Fourier components across time. Using these as inputs in Eq. 1 and solving the latter for stress, the equivalent stiffness and loss tangent may then be readily computed (see ‘Methods’ and Additional file 6: Figure S6).

**Fig. 4:**
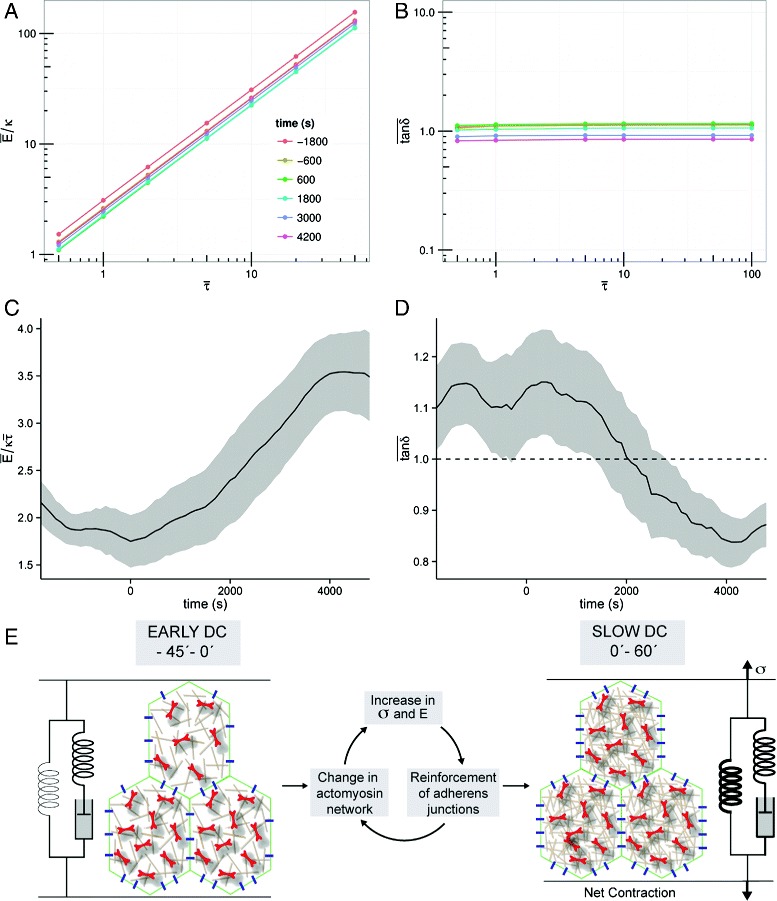
$\bar \tau $ dependence of material parameters and emergent material properties of amnioserosa cells. Average stiffness **a** and loss tangent **b** for different values of $\bar \tau $ within its estimated physiological range. The data are averaged over all cell cycles within the time slices indicated by the colour legend. **c** Stiffness and **d** loss tangent vs. time averaged over 100-s bins. Stiffness is shown in units of $\kappa \bar \tau $ and the *shaded area* corresponds to 95 % confidence intervals. The time evolution shows that amnioserosa cells become stiffer and more solid-like as dorsal closure progresses. **e** Schematic of the transition during dorsal closure (*DC*) from a fluid-like apicomedial sheet with no net contraction to a net contractile solid-like sheet. This transition is accompanied by increases in effective mechanical properties: a doubling of stiffness (*E*) and a quadrupling of stress (*σ*). The apicomedial material is shown to have emergent properties of a viscoelastic solid, and can usefully be compared to a standard linear solid, in which the fluid-like spring and dashpot in series dominate early dorsal closure, after which the solid-like spring in parallel progressively dominates

With our constitutive equation, we anticipate that the estimates for stress and, consequently, for stiffness and loss tangent will depend on three parameters that cannot be inferred from the present data: the bulk cell elastic modulus *κ*, the stress relaxation times *τ*_R_ and the stress-to-active-force relaxation timescales ratio $\bar \tau $. The first two parameters set units of force and time. As we are interested in the relative, rather than absolute, changes in cell stress as dorsal closure progresses, we can keep these two parameters free. The only parameter value we thus need to specify is that of $\bar \tau $. While we cannot determine a specific value for the latter from our data, we can use reported values of stress relaxation and myosin contractility timescale in the literature [9, 34] to establish that its value must lie within the physiological range of $\bar \tau \in \left [0.1,100\right ]$. Performing a scan to obtain measurements of the material parameters and their time evolution for different values within this physiological range reveals that the loss tangent is largely insensitive to $\bar \tau $, while stiffness exhibits a linear dependence (Fig. 4a, b). This scaling behaviour implies that specifying a value of $\bar \tau $ within its allowed range is irrelevant for the relative evolution of the material parameters. Without loss of generality, we have thus set $\bar \tau = 1$.

By computing stiffness and loss tangents for all cell tracks in our dataset, grouping the data in 100-s intervals and averaging over time, we obtained the evolution of the amnioserosa emergent material parameters across dorsal closure. Our results show a significant change in amnioserosa material properties over time. We observe an increase in stiffness as dorsal closure progresses (Fig. 4c), with values nearly doubling from *t*≃0 s onwards until reaching an apparent plateau at *t*≃4000 s. The loss tangent exhibits a decrease over time (Fig. 4d), implying amnioserosa cells become more solid-like, in agreement with [22]. We note, however, that for most of early to slow dorsal closure (*t*<1800 s) tan*δ* lies above the threshold value of unity. This threshold value marks the transition from liquid-like, viscous-dominant behaviour (tan*δ*>1) to solid-like, elastic-dominant behaviour (tan*δ*<1). While amnioserosa cells exhibit a solid-like response for late dorsal closure, we thus observe that amnioserosa cells behave in time predominantly as a viscoelastic liquid (Fig. 4e).

To validate our analysis, we made use of a mutant situation in which we increase myosin activity in the amnioserosa through the ectopic expression of a constitutive active form of myosin light chain kinase (ctMLCK). It has been previously shown that in these embryos, amnioserosa cells undergo premature apical contraction [19, 35] but their rate of contraction is significantly slower than wild-type cells [10]. Previous laser ablation experiments suggested that these cells are more solid-like compared to the wild type [10]. We thus performed time-lapse movies during the early stages of dorsal closure of ASGal4/UAS-ctMLCK embryos carrying the same membrane and myosin reporters as our wild-type embryos, measured apical cell area changes and myosin fluorescence intensity levels, and used the framework presented above to extract the stiffness and loss tangent for these cells (Additional file 7: Figure S7A–E). For a similar developmental stage, we find that stiffness is higher in ASGal4/UAS-ctMLCK embryos compared to wild type, while loss tangent is lower, revealing that these cells are more solid-like and also stiffer (Additional file 7: Figure S7F). These results are consistent with laser ablation data [10] and with the known effect of myosin phosphorylation on the mechanical properties of cytoskeletal networks in other contexts [36]. This analysis provides further support to our theoretical framework, allowing us to extract unambiguous mechanical properties of cells undergoing oscillatory behaviour in different mutant backgrounds.

### Tissue stress evolution

Having obtained estimates for the relative change in stiffness and loss tangent over 7000 s of dorsal closure in wild-type embryos, we made use of previous laser ablation studies [22] to shed further light on the evolution of relative tissue stress. In that study, ablations were performed either at cell junctions or in the medial region. Relative stress estimates were obtained by relating the strain of the edge of the laser ablated region to cell stress via the hysteretic damping law:

(3) $$ \epsilon (t) = \frac{\sigma}{G_{0}} \left(\frac{t}{t_{0}}\right)^{2\delta/\pi},  $$

which describes the linear response of strain to a unit step change in stress. Here, *G*_0_ and *t*_0_ are scale factors for stiffness and time, and *δ* is the loss angle, all of which are assumed to be independent of the location of ablation. As the scale factors could not be determined in [22], the authors could only obtain relative, unscaled stress measurements: $\Delta = \sigma /(G_{0} t_{0}^{2\delta /\pi })$. Adapting the hysteretic damping law to the case of sinusoidal loading, we can straightforwardly obtain an equation for stiffness *E* in terms of the loss angle *δ* and the scale factors *G*_0_ and *t*_0_ [37]. Using this equation (Eq. 10) together with the values for the stiffness and loss tangent obtained in the previous section, we can then estimate *G*_0_ and *t*_0_ with a non-linear fit. Combining these estimates with the measurements for *Δ* reported in [22], we can therefore calculate the relative change in stress as dorsal closure progresses. The two time points sampled in [22], stage 13 and stage 14, correspond approximately to 1000 and 4000 s in our analysis, so we calculated the fold-change in stress over this period (Table 1). We find that medial stress increases about fourfold, twice as much as the increase in both cell stiffness (Fig. 4c) and junctional stress.

**Table 1:** Cell stiffness and estimates of stress evolution between slow (stage 13, *t*≃1000 s) and fast (stage 14, *t*≃4000 s) phases of dorsal closure (± standard error)

Cell site	Relative stiffness	Relative stress
	*E*_14_/*E*_13_	*σ*_14_/*σ*_13_
Medial	1.76±0.27	3.99±1.34
Junctional		1.94±0.71

## Discussion

Measuring forces and stresses in living tissues in a systematic and comprehensive manner remains an important challenge in the field of animal morphogenesis. Recently, methods to infer forces from cell shapes and their deformations have been implemented that assume cell pressure and junctional tension to be the dominant contributors determining cell shape [14–16]. We provide here an alternative and complementary framework for measuring emergent mechanical properties in vivo and in a non-invasive manner (see information flow summary in Additional file 6: Figure 6), that is suited to tissues in which the main active force is oscillatory and exerted across the apicomedial surface of cells.

In this work, we have made use of high-throughput data of cell shape changes and myosin fluorescence intensity levels of hundreds of cells over the course of 2 hours of embryonic development. These data allowed us to analyse the dynamics of myosin with unprecedented precision. We have found that the frequency of myosin oscillations increases during dorsal closure in the same manner as apical cell area oscillations. However, in contrast to the observed decrease in the amplitude of apical cell area oscillations, the amplitude of myosin oscillations remains constant during most of dorsal closure. Moreover, the average lag between the myosin fluorescence intensity and the apical cell area signals decreases during the course of the process. These observations led us to investigate the evolution of material properties of amnioserosa cells. For this, we established a constitutive equation for the amnioserosa that describes the tissue as a linear contractile viscoelastic material. We present evidence that this is a good first approximation of a description of the behaviour of the tissue at timescales relevant for the observed cell behaviour and morphogenesis.

We show for the first time that the apical plane of an intact embryonic epithelium evolves from a fluid-like behaviour to a viscoelastic solid behaviour. Effective tissue stiffness doubles during this transition, at the same time as net contraction of the tissue starts in earnest. Combining our results with previous laser ablation data [22], we can now provide important information on the evolution of stresses in amnioserosa cells. We find that medial stress increases fourfold during the course of dorsal closure while junctional stress increases twofold. Our results reveal that an increase in stress is accompanied by an increase in stiffness. However, at the medioapical region the increase in stress overtakes the increase in stiffness by twofold. These results lead us to suggest that in amnioserosa cells, effective contractile stresses are mostly generated by the apicomedial region and thus that the apical contraction of the tissue is dominated by the apicomedial actomyosin network and not by the junctional actomyosin belt.

Experiments in in vitro reconstituted cytoskeletal networks can aid the mechanistic understanding of the evolution of stress and stiffness in the amnioserosa. In vitro, an increase in myosin motor concentration in cross-linked actin networks increases the stiffness of the network and lowers its loss tangent [36]. This effect is not mediated by the cross-linking activity of myosin but by its motor activity [36]. This is very similar to what we observe in amnioserosa cells upon ectopic expression of a constitutive active form of MLCK, which increases the levels of phosphorylated myosin (this work). Our previous laser ablation experiments have also shown that increasing myosin phosphorylation levels induces cells to become more solid-like [10]. Interestingly, both laser ablation experiments and the rheological analysis presented here show that the amnioserosa tissue of ASGal4/UASctMLCK embryos is around 30 % more solid-like than the wild type at early stages of dorsal closure. We observe that there is a similar decrease of 30 % in amnioserosa loss tangent as dorsal closure progresses. Thus, an interesting hypothesis is that the levels of phosphorylated myosin increase during dorsal closure. Measurements of myosin levels in amnioserosa cells in early and late dorsal closure embryos suggest that there is an increase in apical myosin motor concentration over the course of dorsal closure [19]. A continuous measurement of cellular myosin levels over dorsal closure would be informative but such measurements are hindered by the dynamic nature of the myosin signal, photobleaching and possible changes in its turnover rate [31].

An increase in myosin motor concentration or myosin phosphorylation levels may not be the only mechanism underlying the evolution of the mechanical properties of amnioserosa cells. We have recently shown that amnioserosa cells with increased actin linear polymerisation also become more solid-like and probably stiffer than wild-type cells [10]. Also, there is increasing evidence that adherens junctions mediate the emergence of tissue stiffness and tension in epithelial monolayers [9, 38] and we have previously found an association between tissue stiffness and the stabilisation of E-cadherin at cell–cell junctions [10]. Our preliminary results (data not shown) suggest that adherens junctions are also stabilised as dorsal closure progresses suggesting that the maturation of adherens junctions, and their ability to transmit tension, could also be underlying the observed changes in amnioserosa material properties over time. Alternatively, it is possible that the evolution of the mechanical properties of amnioserosa cells is associated with a decrease in cell volume, as has recently been shown in amnioserosa cells during later stages of dorsal closure [39].

Our results show that an increase in contractile stress is coupled to an increase in effective stiffness, but that the relative increase in stress can exceed that of stiffness. The onset of apical contraction through the engagement of a clutch between cell–cell junctions and a contractile cortex [40], or the attenuation of an actomyosin-aPKC negative feedback loop giving rise to more persistent actomyosin networks [27], or an increase in the density of cytoskeletal elements through a reduction in cell volume [39], could all in principle produce an increase both in stress and stiffness. Whatever the initial stimulus that starts the changes in material properties of the amnioserosa leading to net contraction, it is likely that other changes will follow immediately, making it very difficult to disentangle cause and effect. For example, contractile stresses can strengthen E-cadherin bonds [41], can reinforce adherens junctions through the *α*-catenin-mediated recruitment of vinculin [42] and can recruit and stabilise myosin [43, 44]. Also, two mechanically gated ion channels have been found to have a role in amnioserosa morphogenesis [45], suggesting that both direct and indirect (perhaps with a time lag) tension-mediated effects on cell behaviour could be important. Interestingly, recent results show that cells can increase force production in response to a stiffer environment [13] and that cells are actively mechanoresponsive [33], suggesting that an external mechanical cue may be sufficient to initiate net contraction. The identification of the mechanical feedback mechanisms operating cell-autonomously, through neighbours and at the tissue level, will undoubtedly improve our understanding of the self-organising mechanics of actomyosin systems and how these effect morphogenetic change.

## Conclusions

With non-invasive methods applied to a simple epithelium in vivo, and using myosin fluorescence intensity as a proxy for active force generation, we have been able to quantify relative changes in individual effective mechanical properties. Our results reveal that increases in stress and stiffness are coupled. We propose that in amnioserosa cells, apical contraction is unlikely to be driven primarily by contraction at cell–cell junctions where stress and stiffness increase by the same relative amount. Instead, apical contraction is dominated by the medioapical region where the relative increase in stress is greater than that of stiffness (Fig. 4e). Our results also provide important quantitative information for the generation of biophysical models exploring mechanochemical mechanisms of cell and tissue morphogenesis.

## Methods

### Fly strains and live imaging

For the live imaging, we used a stock carrying ubiECad-GFP [46] and zipperCPTI002907 (available from Kyoto Stock Center). Stage 12–13 *Drosophila* embryos were dechorionated, mounted in coverslips with the dorsal side glued to the glass and covered with Voltalef oil 10S (Attachem). The amnioserosa was imaged at 25–28 °C. using an inverted LSM710 laser scanning microscope with a 63 × oil immersion Plan-Apochromat (NA = 1.4) objective. A region of the amnioserosa was imaged with an argon laser and the emitted signal between 495 and 620 nm was collected using spectral detectors. Five or six *z* sections 1 *μ*m apart were collected every 10 s. Signals specific to green fluorescent protein and yellow fluorescent protein were extracted using the linear unmixing tool.

The resulting confocal images were then segmented in a semi-automated manner using custom software written in Interactive Data Language (IDL, Exelis) and described in [19, 28]. Individual embryos (*N*=23) were staged according to three parameters, which have been shown to evolve stereotypically through the course of dorsal closure [24]: cell area, cell shape anisotropy and mediolateral cell length. This allowed us to determine their developmental time with an accuracy of 10 min (Additional file 1: Figure S1, Additional file 2: Figure S2). Imaged embryos were left to develop until larval stages to check that they survive normally until the end of embryogenesis.

### Data analysis and statistics

The data were analysed using custom code written in R [47] with the *data.table* [48] and *plyr* [49] packages. The filtering procedure made use of the packages *signal* [50] and *seewave* [51]. Plots were produced using the *ggplot2* package [52]. Unless otherwise indicated, confidence intervals were calculated via a hierarchical bootstrap, with embryo ID and cell label as clustering factors [53, 54]. Strain-to-myosin coherence confidence intervals were calculated by computing, for every time step *t*_*i*_ and at *N*=1000 iterations per step, the myosin signal of a randomly sampled cell and the strain signal of a sampled cell present at *t*_*i*_.

### Computing myosin and strain

The raw medial myosin fluorescence intensity of individual cells was calculated as the total pixel fluorescence intensity excluding a zone of 3 pixels in width surrounding cell edges defined by the membrane signal. To compare reliably the myosin fluorescence signal across different embryos, different cells and throughout developmental time, we require a derived quantity for fluorescence intensity, which is invariant under rescaling of background auto-fluorescence and baseline fluorescence levels. We account for these effects by first subtracting, for every embryo and time-slice, the background fluorescence contribution defined as the peak of the pixel intensity distribution at that time-slice. For each cell track, the background subtracted myosin signal was then divided by the low-frequency myosin trend representing the myosin background and defined as the lower envelope of the myosin signal smoothed over a moving window of 6 min in length. We observe that removing the background fluorescence from the signal before performing this rescaling significantly reduces the intra-embryo and intra-stage variability of relative myosin fluorescence intensity (Additional file 8: Figure S8). Apical area strain was defined as cell area relative to a cell area trend computed using a moving window of 6 min. High-frequency noise, residual low-frequency and neighbour effects in both area and rescaled myosin signals were removed via a bandpass filter in the range 1 mHz≤*ν*≤12.5 mHz. Filtering was implemented via a forward-backward filter with finite impulse response based on a Hann window [55]. Note that the resulting signals have frequencies far below the Nyquist limit (50 mHz). We have tested other algorithms for strain computation, rescaled myosin calculation as well as other windows for the construction of the filters, and they do not significantly affect our results.

### Fourier analysis

The Fourier decomposition of the strain and myosin signals of individual cells was performed at a frequency resolution of *δν*=0.195 mHz within the range 1 mHz≤*ν*≤12.5 mHz. To track the time evolution of each of the Fourier components, we used a sliding window with the frequency-dependent length 3/*ν* together with a Hanning taper [55]. The step size of the sliding window at each frequency was set to 1/2*ν*. Oscillation amplitudes were calculated as the maximum absolute values of the decomposed signal closest to the centre of the tapering window. The strain-to-myosin lag at each frequency band was obtained by computing the instantaneous phases of the two signals via a Hilbert transform and calculating their phase difference. The strain-to-myosin coherence was computed via a wavelet decomposition using a Morlet mother wavelet.

### Influence of neighbour myosin phase

The myosin phase was determined relative to the previous and subsequent peaks in a cell’s myosin signal. Cells were considered to have fluctuating myosin if the current cycle length was between 1 and 6 min, and if the current myosin amplitude multiplied by frequency was greater than 0.3, discounting data with no or negligible fluctuation. The correlation in myosin phase between immediate neighbours, *r*_m_, was calculated for the 117,798 neighbour pair instances (sampled every 10 s) where both cells had fluctuating myosin, with *r*_m_=1 for a phase difference of zero, and *r*_m_=−1 for a phase difference of *π*. For each cell at each sampled time instance, a two-dimensional cell shape strain rate tensor was calculated that best transformed its current cell shape to that of the next time step, assuming no cell rotation [19, 28]. The cell shape strain rate in the orientation of neighbour pair cell centroids was then extracted as the projection of the tensorial strain rate along this orientation.

### Myosin photobleaching

To verify the presence of photobleaching of the myosin signal, we continuously imaged half of the amnioserosa of an early dorsal closure embryo under the same protocol used in the rest of our data acquisition for 40 min and, at the end of this period, imaged the whole amnioserosa and compared the myosin fluorescence levels of the two halves (Additional file 3: Figure S3). Our experiment revealed a marked decrease in the relative fluorescence levels of the continuously imaged half, in agreement with the decrease in myosin fluorescence minima and consistent with photobleaching.

### Derivation of the constitutive equation

In [33], a derivation is given for a tensorial constitutive equation describing the mechanics of actomyosin, which can be written in the notation of the present paper as:

(4) $$ \boldsymbol{\sigma} + \tau_{\mathrm{R}} \boldsymbol{\dot\sigma} = \frac{\bar\tau \kappa}{2} c_{\mathrm{m}} \boldsymbol{I} + \tau_{\mathrm{R}} \kappa \boldsymbol{\dot\varepsilon},  $$

where ***σ*** is the two-dimensional stress tensor in the plane of apicomedial actomyosin, $\boldsymbol {\dot \varepsilon }$ the corresponding strain-rate tensor and ***I*** the identity tensor. Note that this equation is the linear Maxwell constitutive equation for viscoelastic liquids with an additional term accounting for myosin action. Here, this term is taken to be isotropic (identity tensor ***I***), since myosin distribution is not observed to have a directional bias in the amnioserosa.

At the amnioserosa scale, the strain that is recorded in our experiments has its principal directions along the anterior-posterior (AP) and mediolateral (ML) orientations (see also [28]), thus the rate-of-strain tensor is a diagonal tensor, and we can write:

$$\boldsymbol{\dot\varepsilon} = \left(\begin{array}{cc} \dot\varepsilon_{\text{AP}} & 0 \\ 0 & \dot\varepsilon_{\text{ML}} \\ \end{array} \right), $$

where the diagonal components denote the strain rate along the AP and ML orientations, respectively. Using Eq. 4, we infer that in the AP–ML frame the stress tensor ***σ*** is also diagonal. The symmetry of the amnioserosa in both AP and ML directions across time is consistent with this, as well as its shape evolution after removal of canthi [17]. Observe that since all tensors in Eq. 4 are diagonal in our case, this tensorial equation can be written as two uncoupled scalar equations, in terms of the stress in the AP direction *σ*_AP_ (respectively in the ML direction, *σ*_ML_) and of the strain rate in the AP direction $\dot \varepsilon _{\text {AP}}$ (respectively in the ML direction, $\dot \varepsilon _{\text {ML}}$). Summing these equations, we obtain:

(5) $$ {\sigma} + \tau_{\mathrm{R}} {\dot\sigma} = \bar\tau \kappa c_{\mathrm{m}} + \tau_{\mathrm{R}} \kappa (\dot{\varepsilon}_{\text{AP}}+\dot{\varepsilon}_{\text{ML}}),  $$

where we have defined *σ*=*σ*_AP_+*σ*_ML_. Observing that up to the first order, the area variation rate is $\dot {A}/A = \dot {\varepsilon }_{\text {AP}} + \dot {\varepsilon }_{\text {ML}}$, we obtain Eq. 1.

### Material parameters for linear viscoelasticity

For oscillatory strain and myosin inputs of the form

(6) $$ \varepsilon_{\omega}(t) = \varepsilon_{0} \cos\left(\omega t\right), \quad c_{\mathrm{m},\omega}(t) = c_{0} \cos\left(\omega t + \delta_{\mathrm{m}}\right),  $$

where *ω* is the oscillation frequency, *ε*_0_ and *c*_0_ are the strain and myosin oscillation amplitudes, and *δ*_m_ is the lag between the strain and myosin signals relative to the oscillation period, the solution to Eq. 1 for *σ*(*t*) can be put in the general form of Eq. 2:

(7) $$ \sigma_{\omega}(t) = \mathit{E}(\varepsilon_{0}, c_{0}, \delta_{\mathrm{m}}) \varepsilon_{0} \cos\left(\omega t + \delta(\varepsilon_{0}, c_{0}, \delta_{\mathrm{m}})\right).  $$

Here, *E* and *δ* denote the stiffness and loss angle, respectively, and are given by

(8) $$ \mathit{E} = \frac{\kappa}{\varepsilon_{0}} \sqrt{\frac{{c_{0}^{2}} \bar\tau^{2}\cos^{2}\delta_{\mathrm{m}} + \left(\varepsilon_{0}\bar\omega + c_{0}\bar\tau\sin\delta_{\mathrm{m}} \right)^{2}}{1 + \bar\omega^{2}}},  $$

(9) $$ \delta = \arctan\left[\frac{\varepsilon_{0}\bar\omega - c_{0} \bar\tau\bar\omega \cos\delta_{\mathrm{m}} + c_{0} \bar\tau\sin\delta_{\mathrm{m}}}{\varepsilon_{0}\bar\omega^{2} + c_{0}\bar\tau\cos\delta_{\mathrm{m}} + c_{0}\bar\tau\bar\omega\sin\delta_{\mathrm{m}}}\right],  $$

where we have defined $\bar \omega \equiv \omega \tau _{\mathrm {R}}$ and $\bar \tau \equiv \tau _{\mathrm {R}}/\tau _{\mathrm {m}}$. Inputting our measurements for strain amplitude, myosin amplitude and strain-to-myosin lag for each Fourier component across time into these equations and specifying a value for $\bar \tau $, we can then estimate the material parameters for the amnioserosa cells. The time evolution of the material parameters in Fig. 4 were then obtained by averaging these results over the frequencies and across time. Additional file 9: Figure S9 shows the frequency dependence of the average stiffness and loss tangent across time.

### Estimating the scale factors for hysteretic damping

For sinusoidal loading and assuming linearity, the hysteretic damping law leads to the following equation for the stiffness [37]:

(10) $$ \mathit{E}(\omega) = G_{0} \Gamma\left(1-\frac{2\delta}{\pi}\right) \left(\omega t_{0}\right)^{2\delta/\pi},  $$

where *ω* is the oscillation frequency, *Γ* is the gamma function, *δ* is the loss angle, and *G*_0_ and *t*_0_ are scale factors. Using the values for *E* and *δ* obtained in our analysis of the evolution of the material parameters in dorsal closure, we can estimate *G*_0_ and *t*_0_ via a non-linear fit.

### Data availability

Time-lapse movies are available on request.

## Additional files

Additional file 1
**Figure S1.** Overview of dataset. Individual embryos shown vs. dorsal closure developmental time. The colour code corresponds to the number of individual cell tracks per embryo at each time point. Data beyond the *black dashed line* are excluded from our analysis. (PDF 48.9 kb)

Additional file 2
**Figure S2.** Embryo staging and evolution of staging parameters. Individual embryos staged according to the evolution of three stereotypical parameters in dorsal closure: **A** average amnioserosa cell axial elongation bias (positive is oriented in mediolateral direction), **B** average cell length in the mediolateral direction, and **C** average cell area. Curves for individual embryos, which span on average 40 min of dorsal closure each, were manually aligned against a template curve covering the entire dorsal closure process [24]. The template corresponds to *dashed black lines*. The *grey shaded area* is excluded from our analysis. This staging procedure is accurate to within 10 min. Notice that there is an appreciable variability between embryos. That the average cell area of the embryos analysed is generally above the template curve is because of under-sampling of more marginal cells. This is due to limitations of the imaging procedure, in which for the temporal resolution, we were not able to image the whole amnioserosa but were restricted to the more central region of the tissue. (PDF 1187 kb)

Additional file 3
**Figure S3.** Photobleaching of myosin signal. Photobleaching was estimated by continuously imaging one half of the amnioserosa only (labelled b) for 40 min and comparing myosin intensity levels between the two halves at the end of this interval. We observe an attenuation in the fluorescence intensity of the continuously imaged half (a vs. b). (JPG 123 kb)

Additional file 4
**Figure S4.** Influence of the myosin status of neighbouring cells. **A** The effect of neighbouring cell myosin phase on cell shape strain rate in the orientation of the cell pair centroids, broken down by the correlation of myosin phase between neighbour pairs. The shape strain rate of neighbouring cells with anti-correlated myosin phases is strongly enhanced, whereas it is reduced for neighbours with correlated myosin phase. **B** The data used in Fig. 1 i are represented, but here with neighbouring cells substituted by other cells with myosin fluctuation from the tissue chosen at random. With the exception of a significant but small enhancement of the rate of area change for neighbours with negatively correlated myosin phases (see Fig. 1 i), the randomised data are indistinguishable from the non-randomised data. *Dotted lines* show 95 % confidence intervals in (**A**) and (**B**). (PDF 1024 kb)

Additional file 5
**Figure S5.** Examples of non-linear myosin–strain hysteresis cycles. **A** Cycle for a saturating myosin force, **B** strain-stiffening material and **C** viscoplastic behaviour. (PDF 263 kb)

Additional file 6
**Figure S6.** Sketch of the information flow used to infer emergent properties of amnioserosa. From the observation of strain dynamics and myosin activity, the microstructure model (Eq. 1) predicts the stress. The emergent rheology is then approximated by a linear relation (Eq. 2) between the predicted stress and the observed oscillatory strain, characterised for each developmental time *t* and frequency *ω* by the complex modulus *E* ^∗^(*t*,*ω*)=*E* exp(i*δ*). Averaging over all cells and over a range of frequencies yields the time evolution of representative emergent parameters, the average stiffness $\bar {E}$ and loss tangent $\overline {\tan \delta }$. (PDF 2099 kb)

Additional file 7
**Figure S7.** Live imaging and cell area and myosin measurements of ASGal4/UAS-ctMLCK embryos. **A** Confocal dorsal projections of amnioserosa tissue carrying membrane markers (DECadGFP, *red*) and myosin markers (zipperYFP, *green*) during slow dorsal closure of an example ASGal4/UAS-ctMLCK embryo. **B** Apical area and **D** myosin fluorescence intensity (*f.i.*) evolution for a sample cell in the dataset. The area trend and myosin minima trends are shown in *orange*. Resulting **C** apical strain and **E** rescaled (*resc.*) myosin for the raw signals (**B**) and (**D**), respectively. **F** Summary of stiffness and loss tangent of amnioserosa cells of wild-type and ASGal4/UAS-ctMLCK embryos. For wild-type embryos, the values of stiffness and loss tangent were broken down into four developmental stages as indicated in the figure. They are in arbitrary units (*a.u.*): stiffness wild type −1800–0 s: 1.90±0.21, loss tangent wild type −1800–0 s: 1.12±0.08, stiffness wild type 0–1800 s: 1.99±0.35, loss tangent wild type 0–1800 s: 1.11±0.1, stiffness wild type 1800–3600 s: 2.82±0.46, loss tangent wild type 1800–3600 s: 0.93±0.08, stiffness wild type 3600–4800 s: 3.51±0.43, loss tangent wild type 3600–4800 s: 0.85±0.05. Note that the stiffness and loss tangent shown for ASGal4/UAS-ctMLCK embryos (3.246±0.483 arbitrary units and 0.824±0.049 arbitrary units, respectively) correspond to the 0–1800-s developmental stage. *Bars* indicate 95 % confidence interval of experimental averages. (PDF 2877 kb)

Additional file 8
**Figure S8.** Relative variance of two candidate, scale-invariant variables derived from our raw myosin measurements. *wobs* corresponds to rescaled myosin fluorescence intensity without background subtraction, while *wbs* corresponds to rescaled myosin with background subtraction. We note that the relative variance of wbs myosin at the intra-embryo **A** and intra-stage **B** levels is lower than wobs, motivating the use of the former as our myosin variable. *DC* dorsal closure. (PDF 617 kb)

Additional file 9
**Figure S9.** Spectrograms for **A** average stiffness and **B** loss tangent, obtained by pooling individual cell data at each frequency component into 100-s intervals. (PDF 1413 kb)
