# Supplementary figures and images for: Emergent material properties of developing epithelial tissues
Source: BMC Biol. 2015 Nov 23;13:98. doi: 10.1186/s12915-015-0200-y (PMC4656187; doi:10.1186/s12915-015-0200-y)

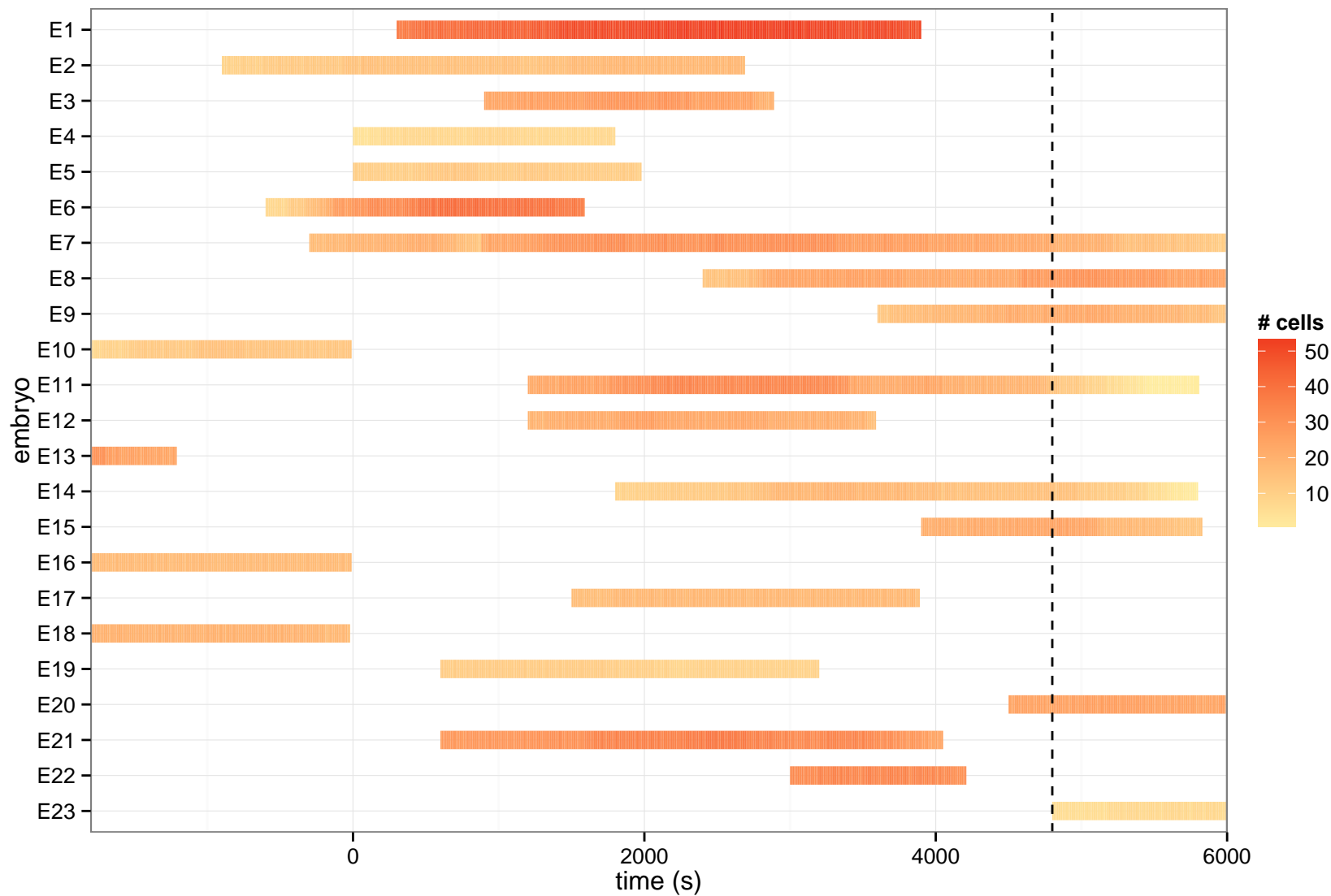

Supplement: Additional file 1 — Figure S1. Overview of dataset. Individual embryos shown vs. dorsal closure developmental time. The colour code corresponds to the number of individual cell tracks per embryo at each time point. Data beyond the black dashed line are excluded from our analysis. (PDF 48.9 kb) [file 12915_2015_200_MOESM1_ESM.pdf]

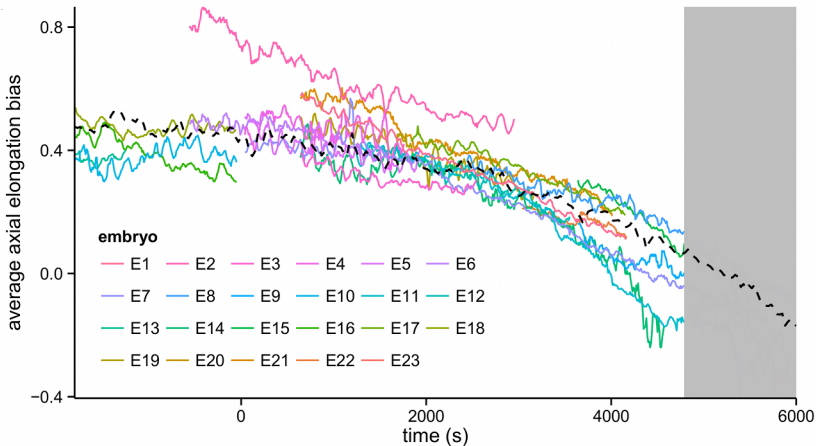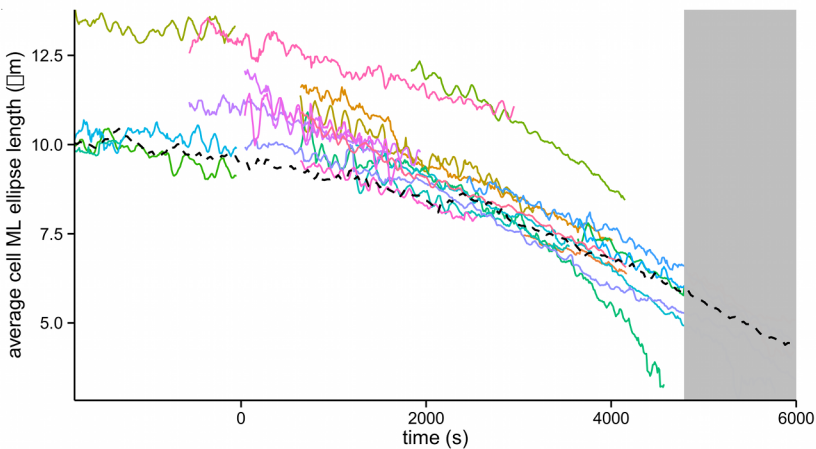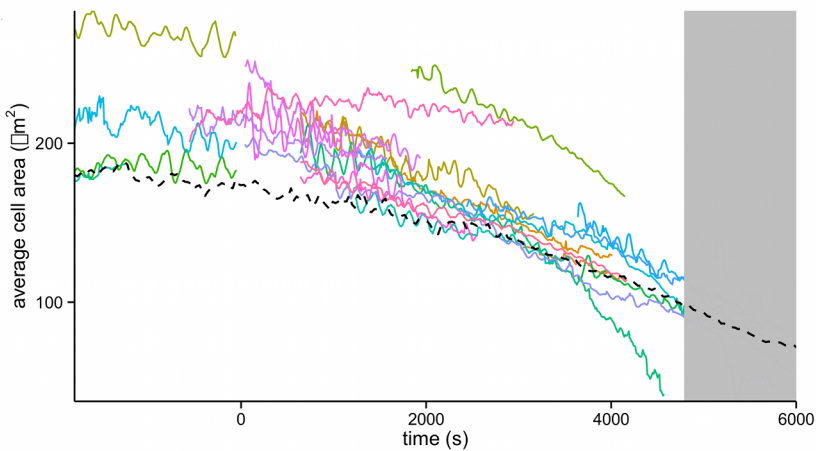

Supplement: Additional file 2 — Figure S2. Embryo staging and evolution of staging parameters. Individual embryos staged according to the evolution of three stereotypical parameters in dorsal closure: A average amnioserosa cell axial elongation bias (positive is oriented in mediolateral direction), B average cell length in the mediolateral direction, and C average cell area. Curves for individual embryos, which span on average 40 min of dorsal closure each, were manually aligned against a template curve covering the entire dorsal closure process [24]. The template corresponds to dashed black lines. The grey shaded area is excluded from our analysis. This staging procedure is accurate to within 10 min. Notice that there is an appreciable variability between embryos. That the average cell area of the embryos analysed is generally above the template curve is because of under-sampling of more marginal cells. This is due to limitations of the imaging procedure, in which for the temporal resolution, we were not able to image the whole amnioserosa but were restricted to the more central region of the tissue. (PDF 1187 kb) [file 12915_2015_200_MOESM2_ESM.pdf]

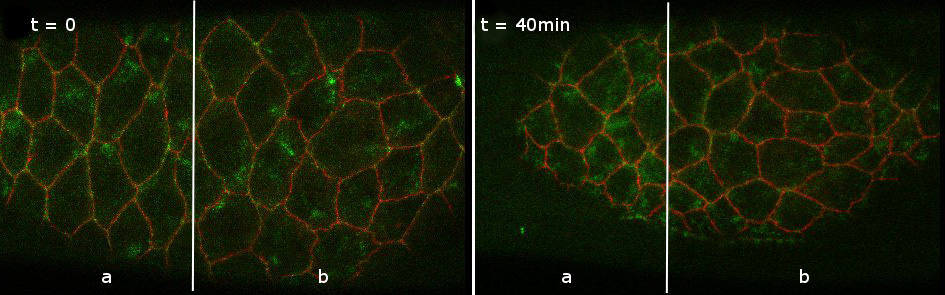

Supplement: Additional file 3 — Figure S3. Photobleaching of myosin signal. Photobleaching was estimated by continuously imaging one half of the amnioserosa only (labelled b) for 40 min and comparing myosin intensity levels between the two halves at the end of this interval. We observe an attenuation in the fluorescence intensity of the continuously imaged half (a vs. b). (JPG 123 kb) [file 12915_2015_200_MOESM3_ESM.jpg]

A

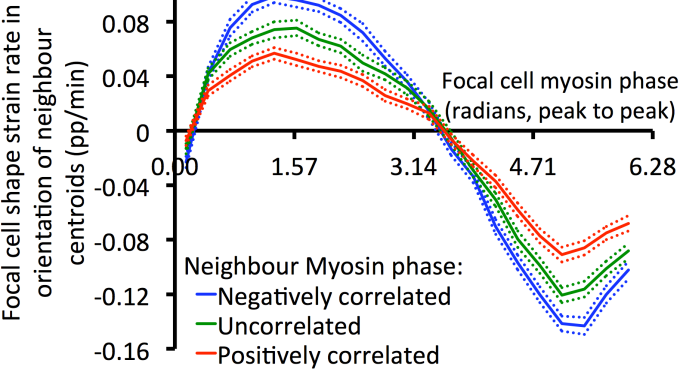

B

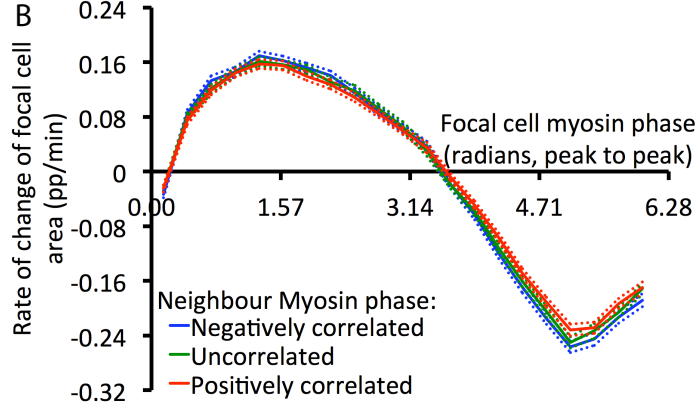

Supplement: Additional file 4 — Figure S4. Influence of the myosin status of neighbouring cells. A The effect of neighbouring cell myosin phase on cell shape strain rate in the orientation of the cell pair centroids, broken down by the correlation of myosin phase between neighbour pairs. The shape strain rate of neighbouring cells with anti-correlated myosin phases is strongly enhanced, whereas it is reduced for neighbours with correlated myosin phase. B The data used in Fig. 1 i are represented, but here with neighbouring cells substituted by other cells with myosin fluctuation from the tissue chosen at random. With the exception of a significant but small enhancement of the rate of area change for neighbours with negatively correlated myosin phases (see Fig. 1 i), the randomised data are indistinguishable from the non-randomised data. Dotted lines show 95 % confidence intervals in (A) and (B). (PDF 1024 kb) [file 12915_2015_200_MOESM4_ESM.pdf]

A

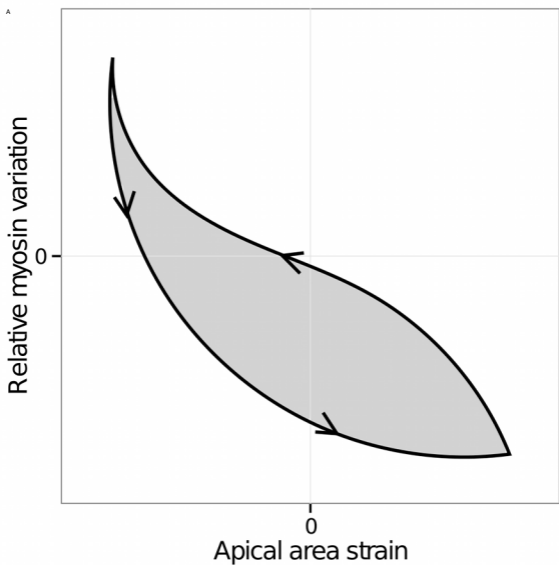

B

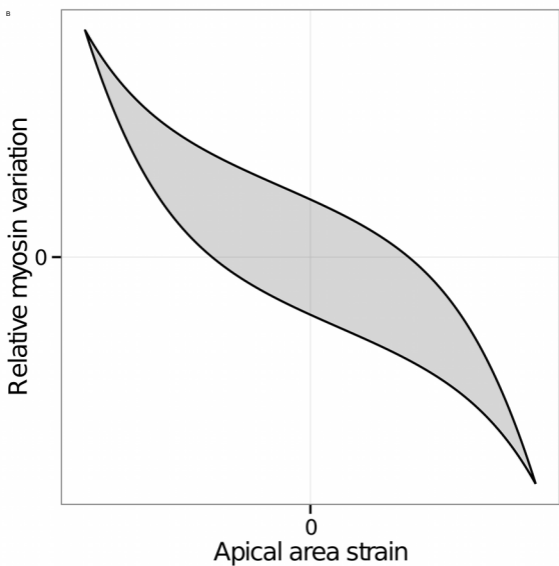

C

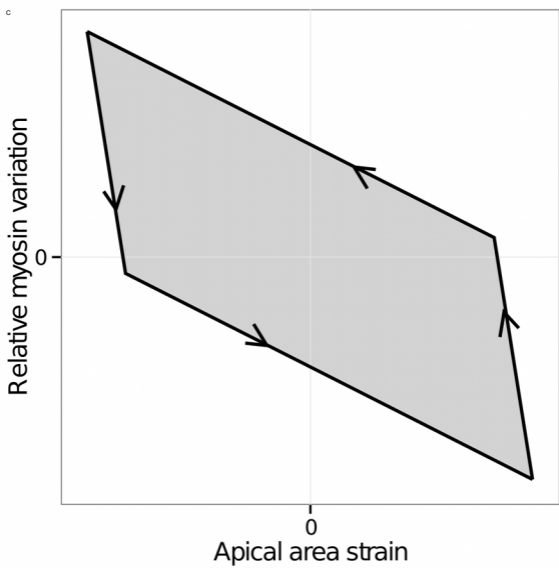

Supplement: Additional file 5 — Figure S5. Examples of non-linear myosin–strain hysteresis cycles. A Cycle for a saturating myosin force, B strain-stiffening material and C viscoplastic behaviour. (PDF 263 kb) [file 12915_2015_200_MOESM5_ESM.pdf]

**A**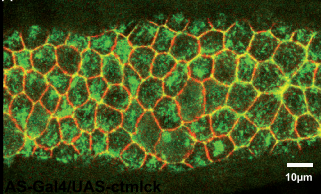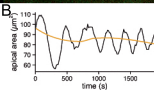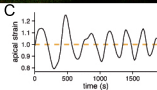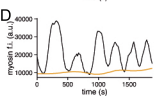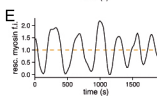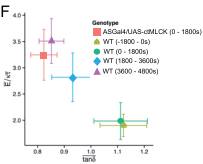

Supplement: Additional file 7 — Figure S7. Live imaging and cell area and myosin measurements of ASGal4/UAS-ctMLCK embryos. A Confocal dorsal projections of amnioserosa tissue carrying membrane markers (DECadGFP, red) and myosin markers (zipperYFP, green) during slow dorsal closure of an example ASGal4/UAS-ctMLCK embryo. B Apical area and D myosin fluorescence intensity (f.i.) evolution for a sample cell in the dataset. The area trend and myosin minima trends are shown in orange. Resulting C apical strain and E rescaled (resc.) myosin for the raw signals (B) and (D), respectively. F Summary of stiffness and loss tangent of amnioserosa cells of wild-type and ASGal4/UAS-ctMLCK embryos. For wild-type embryos, the values of stiffness and loss tangent were broken down into four developmental stages as indicated in the figure. They are in arbitrary units (a.u.): stiffness wild type −1800–0 s: 1.90±0.21, loss tangent wild type −1800–0 s: 1.12±0.08, stiffness wild type 0–1800 s: 1.99±0.35, loss tangent wild type 0–1800 s: 1.11±0.1, stiffness wild type 1800–3600 s: 2.82±0.46, loss tangent wild type 1800–3600 s: 0.93±0.08, stiffness wild type 3600–4800 s: 3.51±0.43, loss tangent wild type 3600–4800 s: 0.85±0.05. Note that the stiffness and loss tangent shown for ASGal4/UAS-ctMLCK embryos (3.246±0.483 arbitrary units and 0.824±0.049 arbitrary units, respectively) correspond to the 0–1800-s developmental stage. Bars indicate 95 % confidence interval of experimental averages. (PDF 2877 kb) [file 12915_2015_200_MOESM7_ESM.pdf]

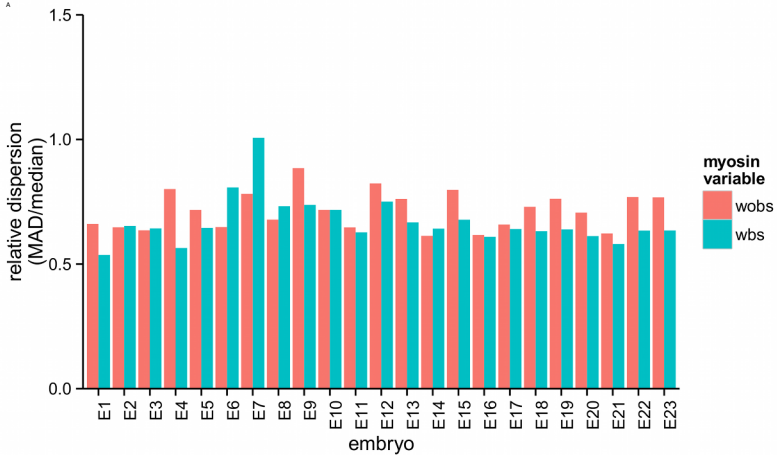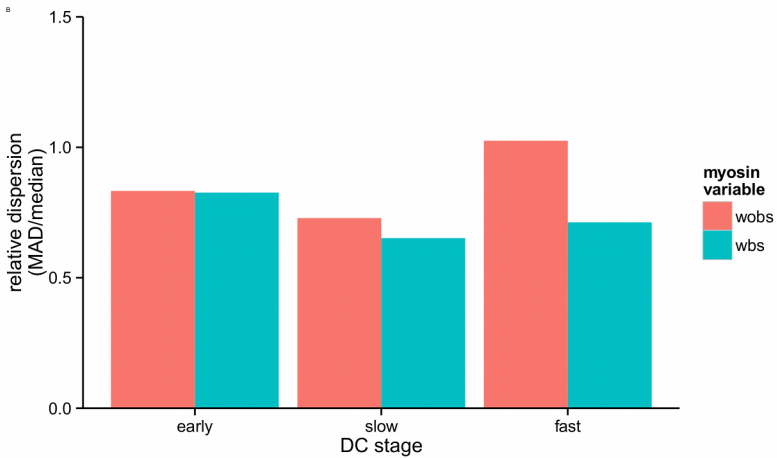

Supplement: Additional file 8 — Figure S8. Relative variance of two candidate, scale-invariant variables derived from our raw myosin measurements. wobs corresponds to rescaled myosin fluorescence intensity without background subtraction, while wbs corresponds to rescaled myosin with background subtraction. We note that the relative variance of wbs myosin at the intra-embryo A and intra-stage B levels is lower than wobs, motivating the use of the former as our myosin variable. DC dorsal closure. (PDF 617 kb) [file 12915_2015_200_MOESM8_ESM.pdf]

A

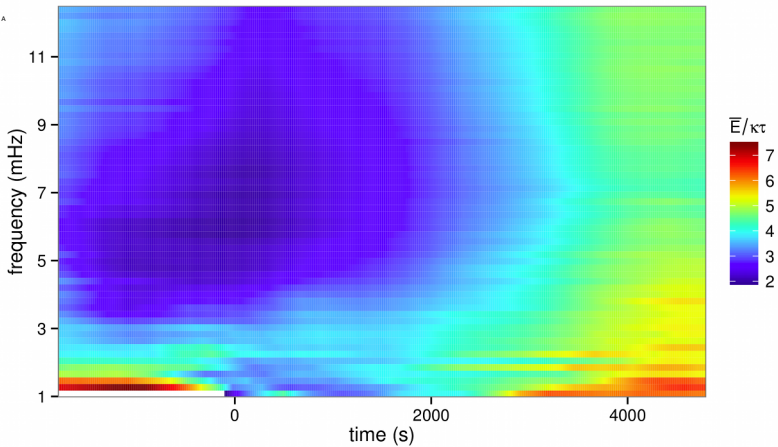

B

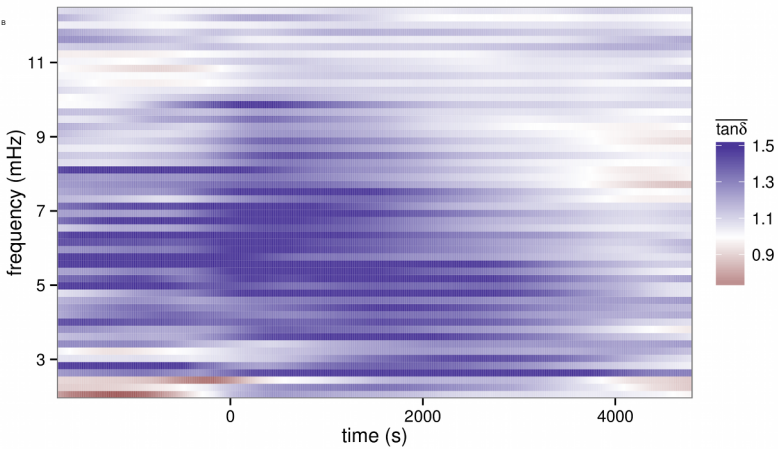

Supplement: Additional file 9 — Figure S9. Spectrograms for A average stiffness and B loss tangent, obtained by pooling individual cell data at each frequency component into 100-s intervals. (PDF 1413 kb) [file 12915_2015_200_MOESM9_ESM.pdf]
