# Supplementary material for: Emergent material properties of developing epithelial tissues
Source: BMC Biol. 2015 Nov 23;13:98. doi: 10.1186/s12915-015-0200-y (PMC4656187; doi:10.1186/s12915-015-0200-y)
Supplement: Additional file 6 — Figure S6. Sketch of the information flow used to infer emergent properties of amnioserosa. From the observation of strain dynamics and myosin activity, the microstructure model (Eq. 1) predicts the stress. The emergent rheology is then approximated by a linear relation (Eq. 2) between the predicted stress and the observed oscillatory strain, characterised for each developmental time t and frequency ω by the complex modulus E ∗(t,ω)=E exp(iδ). Averaging over all cells and over a range of frequencies yields the time evolution of representative emergent parameters, the average stiffness $\bar {E}$Ē and loss tangent $\overline {\tan \delta }$tanδ¯. (PDF 2099 kb) [file 12915_2015_200_MOESM6_ESM.pdf]

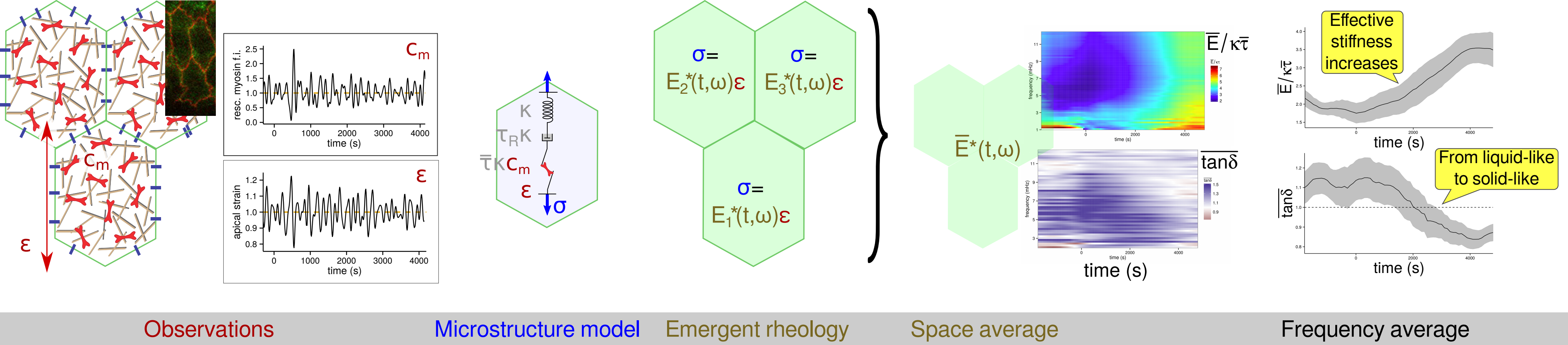

cm
ε
resc. myosin f.i.
time (s)
apical strain
κ
τRκ
τ̄κcm
σ
σ=
E2*(t,ω)ε
E3*(t,ω)ε
E1*(t,ω)ε
Ē*(t,ω)
Ē/κτ̄
tanδ
time (s)
Effective stiffness increases
From liquid-like to solid-like
Observations
Microstructure model
Emergent rheology
Space average
Frequency average
